# Gamma Radiation Induced Oxidation and Tocopherols Decrease in In-Shell, Peeled and Blanched Peanuts

**DOI:** 10.3390/ijms13032827

**Published:** 2012-03-02

**Authors:** Adriano Costa de Camargo, Thais Maria Ferreira de Souza Vieira, Marisa Aparecida Bismara Regitano-D’Arce, Severino Matias de Alencar, Maria Antonia Calori-Domingues, Solange Guidolin Canniatti-Brazaca

**Affiliations:** 1 Center for Nuclear Energy in Agriculture, University of São Paulo (CENA/USP), Av. Centenário 303, P.O. Box 96, 13400-970, Piracicaba, SP, Brazil; 2 Department of Agri-Food Industry, Food & Nutrition, “Luiz de Queiroz” College of Agriculture (ESALQ/USP), University of São Paulo, Av. Pádua Dias 11, P.O. Box 9, CEP 13418-900, Piracicaba, SP, Brazil; E-Mails: tvieira@usp.br (T.M.F.S.V.); marisadarce@usp.br (M.A.B.R.D.); smalencar@usp.br (S.M.A.); macdomin@usp.br (M.A.C.-D.); sgcbraza@usp.br (S.G.C.-B.)

**Keywords:** gamma radiation, storage, lipid oxidation, tocopherol contents

## Abstract

In-shell, peeled and blanched peanut samples were characterized in relation to proximate composition and fatty acid profile. No difference was found in relation to its proximate composition. The three major fatty acids were palmitic acid, oleic acid, and linoleic acid. In order to investigate irradiation and storage effects, peanut samples were submitted to doses of 0.0, 5.0, 7.5 or 10.0 kGy, stored for six months at room temperature and monitored every three months. Peanuts responded differently to irradiation, particularly with regards to tocopherol contents, primary and secondary oxidation products and oil stability index. Induction periods and tocopherol contents were negatively correlated with irradiation doses and decreased moderately during storage. α-Tocopherol was the most gamma radiation sensitive and peeled samples were the most affected. A positive correlation was found among tocopherol contents and the induction period of the oils extracted from irradiated samples. Gamma radiation and storage time increased oxidation compounds production. If gamma radiation is considered an alternative for industrial scale peanut conservation, in-shell samples are the best feedstock. For the best of our knowledge this is the first article with such results; this way it may be helpful as basis for future studies on gamma radiation of in-shell crops.

## 1. Introduction

Crude peanuts and its by-products are of great importance in foods worldwide and are ingredients of many recipes. Peanut food products are usually used by consumers from a range of economic statuses. However, potential mycotoxic fungi [1] in peanuts and peanut products are a problem faced by the industry. In addition, peanuts are known to be a source of allergenic proteins, a problem that manifests itself most often in children but also in adolescents and adults [2].

Irradiation of peanuts at 5.0 kGy [3] and 10.0 kGy [4] has been found to be an effective treatment to completely eliminate potentially aflatoxigenic fungi in peanuts. According to Kilcast [5], ionizing radiation is, by definition, sufficiently high in energy to remove an electron from water, which is the main component of foods and living organisms, and to create highly reactive species, including free radicals such as the hydroxy radical, and hydrogen peroxide. The authors also suggested that the predominant useful effects of irradiation rely on reaction of these species with the DNA of microorganisms, causing death. In addition, Oh *et al*. [6] suggested that gamma radiation may reduce the allergenicity of peanut extracts. The authors reported that allergenic proteins that were exposed to irradiation presented distinct structural modifications as a result of aggregation, fragmentation, and the modification of amino acids, which, in turn, affected the solubility of proteins, their tertiary and secondary structure, and their immunogenicity. Alteration of epitopes by denaturation of the peanut, after irradiation, might have induced a lower response by T cells. The allergic reaction appears to be the result of a T_H_2-type T-cell response to one or more common environmental allergens [7]. As mentioned before [5], irradiation causes molecular changes, among which the formation of free radicals is one of the most important for foods with a high fat content. The model proposed by Farmer *et al*. [8] shows the formation of free radicals as the initial step in the mechanism of lipid autoxidation.

In peanut and its products, the ratio of oleic and linoleic fatty acids (O/L) is used as a quality score; the higher the ratio, the greater the product’s shelf life, due to its higher oxidative stability [9]. Recently, IAC Runner 886 peanut cultivar presented a decrease in their O/L from 1.86 to 1.51 for control and gamma irradiated with 15.0 kGy, respectively [10]. Similar results were reported by Mexis and Kontominas [11]. According to their study, monounsaturated fatty acids were preferentially attacked by oxygen to produce primary and secondary oxidation products as opposed to attack of polyunsaturated fatty acids in gamma irradiated cashew nuts. Palmitic acid is one of major fatty acids in peanuts [12], and oleic acid, in terms of content, has been reported to be inversely proportional to the palmitic acid [13]. IAC Runner 886 peanut cultivar presents high percentage of unsaturated fatty acids [10], for this reason this cultivar is hugely susceptible to oxidation. According to Jensen *et al*. [14], the lipid percentage, fatty acid composition, moisture content, and presence of antioxidants, in addition to the surface structure and porosity of the food product, affect its stability. So, the fatty acid composition is not the only factor that should be taken into account.

The aim of this study was to characterize samples of in-shell, peeled and blanched peanuts (*Arachis hypogaea* L.) cv. IAC-Runner 886 subjected to different doses of gamma radiation (0.0, 5.0, 7.5 or 10.0 kGy) and room temperature storage time in relation to their oxidative status as well as their tocopherol contents.

## 2. Results and Discussion

### 2.1. Proximate Composition

Table 1 shows the proximate composition of the peanut samples. Oil content has an important effect on the sensory characteristic of foods because it contributes to mouth feel and carries flavors and aromas. Peanuts are high oil content foods [15]. There is a huge variation reported in the literature concerning the lipid percentage. Davis *et al*. [16] reported that the lipid percentage of peanuts collected from fields located near Dawson, Georgia State, USA, ranged from 23% (cv. FlavoRunner-458) to 40% (cv. C11-239), while Santos [17] reported that the lipid percentage of peanuts grown in Southeast and Northeast of Brazil, respectively, ranged from 46% (cv. BRS 151 L-7) to 49% (cv. IAC-Tupã).

Non-significant differences were found in relation to the proximate composition of the peanut samples (*p* < 0.05). Although blanching process consists of removing the peanut skins, the process did not change the proximate composition of the oilseed. In fact, that can be explained by the low weight of the peanut skins, in average 2.6% in relation to the peanut weight [18].

The current results are in accordance with the proximate composition of Runner type already reported in the literature. Protein, lipid and ash contents ranged from 22.9 to 23.5%, from 45.4 to 47.9% and from 2.1 to 2.2%, respectively [19]. According to Ng *et al*. [15], genetically modified peanuts presented lipids ranging from 48.4 to 50.1%, protein, from 29.5 to 32.4% and ash ranging from 2.2 to 2.6%. Due to the high lipid content of peanuts and its effect on the shelf life of its products, the present study focused on the effects of gamma radiation and storage time on oxidation effects.

### 2.2. Fatty Acid Composition

Table 2 reports the fatty acid compositions of lipid extracts from initial peanut samples (0.0 kGy; time zero). The oleic to linoleic acid (O/L) ratio is a quality index employed for the determination of genetic peanut characteristics classified as normal, mid-, and high-oleic types, ranging from 1 to 1.5; 1.5 to 9.0, and above 9.0, respectively [9]. The present study was carried out with normal oleic peanuts. There were moderated differences among different samples. This is in good agreement with the findings of Shin *et al*. [9] whose data confirm that fatty acid composition can be different, even within the same cultivar and same harvest year. The enzyme Δ12 desaturase catalyzes the reaction of oleic acid to linoleic acid and the oleic to linoleic acid ratio is controlled by the activity of this enzyme. Furthermore, seed maturity can also influence the fatty acid composition of peanuts [12,13]. Since the present study did not control the production field and harvest, differences among samples cannot be clearly explained. Palmitic acid, oleic acid, and linoleic acid were the major fatty acids. According to the literature [12], the remaining fatty acids, stearic, arachidic, eicosenoic, behenic, and lignoceric acids, normally occur in weight percentages between 0.02 and 4.0%, which in fact agrees with the present study.

Different cultivars may have different fatty acid composition. In a recent study, Shin *et al*. [9] analyzed 151 samples from two year crops and noticed that there was a huge variation in relation to the fatty acid content in samples classified as normal, mid-, or high-oleic. The authors reported that palmitic acid (C16:0) ranged from 5.31%, to 11.49%; stearic acid (C18:0), 1.46% to 4.76%; oleic acid (C18:1, ω9), 44.78% to 82.17%; linoleic acid (C18:2, ω6), 2.85% to 33.92%; arachidic acid (C20:0), 0.87% to 2.18%; gondoic acid (C20:1, ω9), 1.09% to 3.13%; behenic acid (C22:0), 0.73% to 4.37%; and lignoceric acid (C24:0), 0.41% to 2.12%. These data are in agreement with the ones obtained in the current work.

### 2.3. Tocopherols

Table 3 shows the tocopherol concentration of the peanut samples. Initial concentration for all samples were γ > α > δ > β-tocopherol. The presence of natural antioxidants such as tocopherol has been widely studied in peanuts [14,20–24]. Tocopherols appear to be responsible compounds for the oil antioxidant capacity, being negligible the contribution of polyphenols. Only small amount of polyphenols were found in nut oils [20]. On the other hand, small concentration of tocopherols has been found in peanut skins [25], whose antioxidant properties are more related to its high concentration of polyphenols such as condensed tannins.

Jonnala *et al*. [22] reported that tocopherol concentration of peanuts ranged from 14.59 to 16.12, 0.70 to 1.03, 6.90 to 10.62 and, from 4.61 to 4.99 mg/100 g, in relation to α, β, γ and δ-tocopherols, respectively. These results are in accordance with the present study (Table 3). According to Shin *et al*. [24], α and γ-tocopherols were predominant in normal, mid and high oleic Runner cultivars, comprising *ca.* 95% of the total vitamin E present in the kernel. It was also found in the current trial that vitamin E represented *ca.* 95.63, 96.44, and 95.33% in relation to α and γ-tocopherols, for in-shell, peeled and blanched control samples, respectively (Table 3).

The current study demonstrated that gamma radiation caused tocopherol losses in all samples and α-tocopherol was the most affected by the process. Right after the process, α-tocopherol contents decreased by 63.31, 44.2 and 37.63% for peeled, in-shell and blanched peanuts, respectively. The highest sensitivity of α-tocopherol can be related to the antioxidant ranking of individual tocopherols reported by Telegdy Kováts and Berndorfer-Kraszner [26]. According to the authors, between 80 and 120 °C the antioxidant activity is δ > γ > α > β-tocopherol, while between 20 and 60 °C is α > γ > β > δ-tocopherol. Since gamma radiation was performed under 25 °C, α-tocopherol contributed the most to the antioxidant properties, as expected. Similarly to α-tocopherol, right after gamma radiation (5.0 kGy, time zero), γ-tocopherol was affected differently in each sample. γ-Tocopherol decreased by 23.54, 13.99 and 11.88% for peeled, in-shell and blanched peanuts, respectively. Regarding short time effects of gamma radiation (time zero), β-tocopherol concentration started to decrease in peeled samples at 5.0 kGy, while in-shell and blanched samples presented the same behavior at 7.5 and 10.0 kGy, respectively. δ-Tocopherol started to decrease in peeled and in-shell samples at 5.0 kGy and 7.5 kGy, respectively, while blanched samples were not affected by gamma radiation on any time of storage. Furthermore, on the third and sixth months of storage, δ-tocopherol concentration in in-shell samples was not statistically different among control and gamma irradiated samples. According to Kilcast [27], vitamin E is the most radiation-sensitive of the fat-soluble vitamins. Bhatti *et al*. [28] reported loss of tocopherols in peanut oil extracted from gamma irradiated peanuts. According to Lalas *et al*. [29], soybean oil submitted to gamma radiation (3.00 kGy) presented up to 92.3% loss of α-tocopherol.

There was moderated decrease in α, β, γ and δ-tocopherols contents of non-irradiated peeled and in-shell samples during storage, while no difference was found for blanched peanuts (Table 3). Regarding the gamma irradiated samples it is possible to notice that storage affected moderately the tocopherol contents of the samples. In-shell and blanched samples were the least affected. Lavedrine *et al*. [30] reported losses of 29, 28 and 30% in relation to α, γ, and δ-tocopherol on the third month of storage of walnuts under 4 °C. Losses of 24 and 20% in relation to total tocopherols were reported by Chun *et al*. [31] in air and vacuum packaged stored peanuts at 21 °C, respectively. In the present study the shells can play a protective function against photooxidation, which is not possible with peeled and blanched samples. Since the blanching process involves heating, Maillard compounds can be generated. According to Davis *et al*. [21], Maillard compounds present antioxidant properties. The authors submitted runner-type peanuts (cv. Georgia green) to lab-scale roasting at 166 °C, from 0 to 77 min. The lowest loss in α-tocopherol content was noticed in peanut samples submitted to the highest roasting intensity. The authors suggested that the final concentration of vitamin E in roasted peanuts or peanut oil is a balance between heat degradation and indirect heat stabilization via the formation of Maillard reaction products.

The present study showed a negative correlation among gamma radiation and most of the individual tocopherol contents (Table 4). Decreasing of the negative correlation of in-shell and blanched samples was observed during the storage, while the opposite was noticed in peeled samples. Peeled samples showed negative correlation as high as *r* = −0.99 for γ-tocopherols, which means an almost perfect correlation.

**Figure 1:**
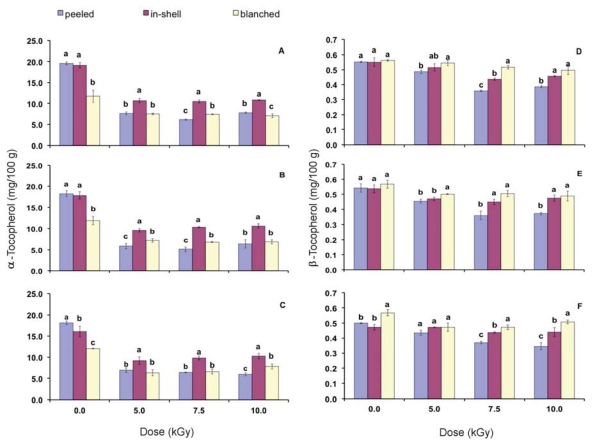
α-Tocopherol content on time zero (**A**), in the third (**B**) and sixth month storage (**C**). β-Tocopherol content on time zero (**D**), in the third (**E**) and in the sixth month storage (**F**). Error bars represent standard deviations of triplicate measurements. Means with the same letter within a gamma radiation dose are not statistically different by Tukey’s multiple test (*p* < 0.05).

**Figure 2:**
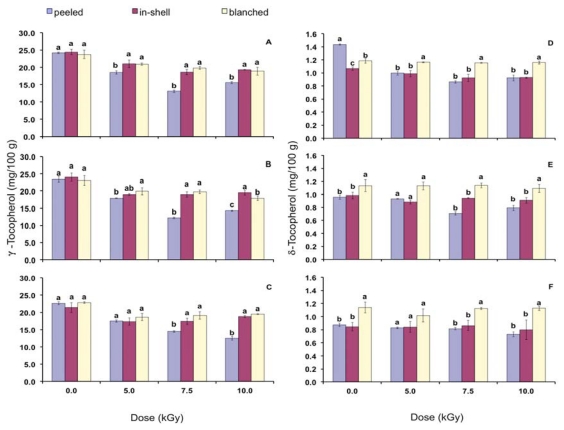
γ-Tocopherol content on time zero (**A**), in the third (**B**) and sixth month storage (**C**). δ-Tocopherol content on time zero (**D**), in the third (**E**) and in the sixth month storage (**F**). Error bars represent standard deviations of triplicate measurements. Means with the same letter within a gamma radiation dose are not statistically different by Tukey’s multiple test (*p* < 0.05).

Figures 1 and 2 show the effect of gamma radiation on α, β, γ and δ-tocopherols with focus on the storage condition. Non irradiated samples (0.0 kGy) on time zero were considered as the control. The ranking for α-tocopherol concentration in control samples was peeled = in-shell > blanched. β and γ-tocopherols did not show differences among the control samples, which demonstrate that the blanching process, that involves the samples heating, did not change their concentration. In control samples the ranking for δ-tocopherol was peeled > blanched > in-shell.

After gamma radiation in-shell samples presented the highest final concentration of α-tocopherol during the whole storage period. In general, the final concentration of β-tocopherols in blanched samples was similar or higher than that of the peeled samples. Initial concentration of α-tocopherol was 19.59 and 11.75 mg/100 g for peeled and blanched samples, respectively, this way the initial concentration for blanched peanuts was 40% less than that of the peeled sample. Peanut samples are submitted to heating to remove their peanut skin. This is probably the reason for the lowest α-tocopherol concentration in blanched peanuts.

Gamma radiation decreased β-tocopherol concentration and blanched samples presented the highest or equal concentration to that of the in-shell samples. In general, peeled samples submitted to gamma radiation have shown the lowest β-tocopherol concentration. No differences were found among non-irradiated samples regarding γ-tocopherol contents during the whole storage period (Figure 2). Right after gamma radiation (time zero) peeled samples presented the highest decrease in their γ-tocopherol content, while no differences were found between in-shell and blanched samples. The ranking of γ-tocopherol contents in gamma irradiated samples was in-shell = blanched > peeled (time zero). On the third and sixth months of storage, at higher doses (7.5 and 10.0 kGy), in-shell samples presented equal or higher γ-tocopherol contents than that of the blanched samples. Gamma irradiated blanched samples presented the highest content of δ-tocopherol and, in general, peeled and in-shell samples were not statistically different from each other.

### 2.4. Oil Stability Index

Gamma radiation decreased the induction period (h) of the crude peanut oil (*p* < 0.05) (Table 5), reducing the oxidative stability of the peanuts. According to Arranz *et al*. [20], crude peanut oil showed induction period of 14.6 h. The longer induction period (higher stability) found by the authors should be due to the lower temperature applied during the analysis (100 °C), which is different from the current study that applied 110 °C to the samples. Even higher damage caused by gamma radiation was reported by Arici *et al*. [32]. According to the authors, cumin oil extracted from gamma irradiated samples presented induction period of 7.72 h (control), 5.43 h (2.5 kGy), 3.60 h (6.0 kGy), 1.92 h (8.0 kGy), and 0.62 h (10.0 kGy). The present study demonstrated that the induction period of gamma irradiated samples was highly correlated to the final (after treatment, after storage) tocopherol content of the samples (Table 6).

The results regarding storage period do not allow the correlation between storage time with induction period of the crude peanut oils. On the contrary of what was expected, the induction period of the blanched peanut oils did not decrease during storage (Table 5). The present results are in good agreement with those of Sanders *et al*. [33]. By means of the analysis of oxidative stability index and peroxide value, the authors showed that the blanching process did not cause reduction in quality of peanuts. According to Cammerer and Kroh [34], with increasing roasting temperature and time, the oxidative stability of peanuts was improved and shelf life prolonged. This can be attributed to the formation of antioxidant Maillard reaction products.

Table 6 shows Pearson’s correlation between gamma radiation and induction period as well as between total tocopherol contents and induction periods.

Negative correlation was found between gamma radiation doses and the induction period. On the contrary, a positive correlation was found between total tocopherol content and induction period. According to Lee *et al*. [35], irradiation with doses up to 5.0 kGy greatly increased oxidation of soybean, cottonseed, and corn oils, as well as linoleic acid. Ascorbyl palmitate was extremely effective at minimizing oxidation in all oils, and its effectiveness was concentration-dependent. Furthermore, ascorbyl palmitate showed significantly greater antioxidative activity than α-tocopherol for the reduction of oxidation in all oils.

**Figure 3:**
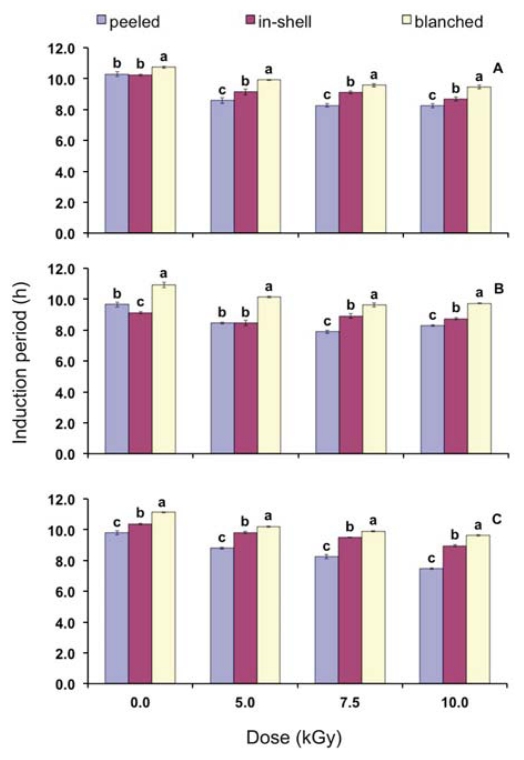
Induction period (h) of oils extracted from peeled, in-shell and blanched peanuts on time zero (**A**), after three (**B**) and six months storage (**C**). Error bars represent standard deviations of triplicate measurements. Means with the same letter within a gamma radiation dose are not statistically different by Tukey’s multiple test (*p* < 0.05).

Since the concentration of tocopherols from the blanched samples was lower than that of peeled and in-shell samples (Table 3), induction periods presented at Figure 3 suggest that this behavior is due to the presence of Maillard compounds, which are known by their antioxidant properties. Maillard compounds probably were generated by the heating during the blanching process. When peeled and in-shell samples are compared with each other, it is possible to notice that, in general, the lowest induction period is attributed to the first one. It suggests that in-shell gamma irradiated peanuts are more stable to termoxidation than peeled peanuts.

### 2.5. UV Absorption

Tables 7 and 8 show, respectively, absorptivity at 232 and 270 nm, indicating the presence of primary (dienes) and secondary oxidation products (aldehydes and ketones). Both primary and secondary oxidation products have had their concentration increased by gamma radiation (*p* < 0.05). The higher the dose the larger was the production of oxidation products.

According to Bhatti *et al*. [28], gamma radiation (8.0 kGy) increased primary and secondary oxidation products of peanuts. Furthermore, the concentrations of the secondary grew faster. The authors reported that gamma radiation increased dienes from 1.51 to 2.69 (cv. Golden) and from 1.71 to 3.25 (cv. Bari). Trienes ranged from 0.11 to 0.51 (cv. Golden) and from 0.12 to 0.63 (cv. Bari). The same behavior was noticed in the present study right after the radiation process (time zero). When control samples are compared to gamma irradiated (10.0 kGy) there was an increase by 125.1% (peeled), 44.7% (in-shell) and 24.6% (blanched) regarding primary oxidation compounds, against 716.7% (peeled); 147.9% (in-shell) and 223.8% (blanched) in relation to the secondary compounds.

Volatile secondary compounds such as aldehydes, ketones and alcohols have had their concentration increased in peanuts, pistachio and cashew nuts submitted to gamma radiation with doses up to 7.0 kGy [11,36], which indicated increase of lipid oxidation. On the third and sixth months of storage there was an increase on the concentration of primary and secondary oxidation products when compared to time zero (Table 7). According to Anwar *et al*. [37], soybean oil stored during six months, under room temperature, had their diene concentration increased from 0.08 to 23.97 and their triene increased from 0.04 to 13.81.

Primarily, due to oxidative reactions of lipids, peanuts shelf life as well as its sensory quality decreases with storage time [34]. According to Jensen *et al*. [14], the light accounted for the greatest systematic variation of the relative levels of free radicals in peanuts, whereas the oxygen availability had the largest influence on the formation of hexanal. In the present study there was oscillation on the concentration of secondary products during the storage. The same was noticed on peanut storage studies from Nepote *et al*. [38]. The volatile nature of the secondary products could be responsible for that oscillation.

Table 9 presents the correlation results between oxidation products and gamma radiation doses. In general there was a positive correlation between doses and production of oxidation compounds.

**Figure 4:**
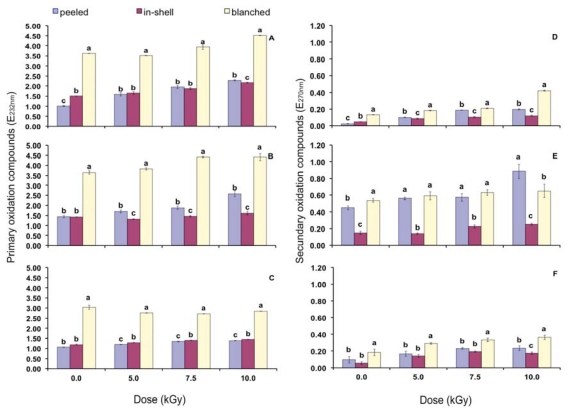
Primary oxidation products on time zero (**A**), after three (**B**) and six months storage (**C**). Secondary oxidation products on time zero (**D**), after three (**E**) and six months storage (**F**). Error bars represent standard deviations of triplicate measurements. Means with the same small letter within a gamma radiation dose are not statistically different by Tukey’s multiple test (*p* < 0.05).

Figure 4 presents the effect of gamma radiation on the oxidation compounds with focus on the storage condition. According to Mexis *et al*. [39], aldehydes such as acetaldehyde, hexanal, nonanal and decanal as well as ketones such as 2-butanone and 2-propanone were formed in almond kernels especially at higher doses (7.0 kGy) as a result of lipid oxidation due to irradiation.

In general, the presence of primary and secondary oxidation products was higher in blanched samples, irradiated or not. In relation to the secondary compounds it is clear that in-shell peanut samples were the less damaged by gamma radiation. During the whole storage, even at higher doses, in-shell gamma irradiated peanuts presented lower secondary oxidation compounds than that of the non-irradiated blanched peanuts. Commercialization of blanched peanuts is already done successfully. In turn, if the presence of volatile compounds is considered as a rejection issue by the consumers it is possible to suggest that there is a small chance in-shell gamma irradiated peanuts may be rejected by them.

## 3. Experimental Section

### 3.1. Material

Samples of in-shell, peeled and blanched cv. IAC-Runner 886 (crop year 2009/2010) were obtained from CAP—Agroindustrial, Dumont, São Paulo State, Brazil.

### 3.2. Methods

#### 3.2.1. Irradiation Process

In-shell, peeled and blanched peanut samples were separated into 1.5 kg portions and placed in polyethylene plastic bags. The bags were irradiated at doses of 0.0, 5.0, 7.5, and 10.0 kGy at a dose rate of 7.5 kGy/h. Irradiation process was carried out in the city of São Paulo, São Paulo State, Brazil, using a multipurpose Cobalt-60 γ-irradiation facility from Nuclear Energy Research Institute (IPEN). IPEN is an autarchy, associated to the University of São Paulo—supported and operated technically and administratively by the National Nuclear Energy Commission (CNEN). The samples were irradiated under air atmosphere and room temperature (25 °C). The samples were stored for six months at room temperature and analyses were performed at the beginning of the study and after three and six months of storage. The storage temperature was monitored by thermo-hygrometer (RH520A, Extech Instruments, Nashua, NH, USA) and ranged from 22.80 to 28.98 °C during the experiment.

#### 3.2.2. Proximate Composition

Ash, lipids, protein, and fiber and carbohydrates in peanut samples were determined according to AOAC methods [40]. Total carbohydrates were calculated by difference.

#### 3.2.3. Oil Extraction

For analysis of the lipid fraction of peanuts, samples were cold pressed with a Carver Press (Carver, Inc., Wabash, IN, USA). After pressing, crude peanut oil samples were filtered and transferred to amber bottles, nitrogen was injected and the oil samples were frozen (−18 °C) until analysis.

#### 3.2.4. Fatty Acid Composition

Peanut crude oils were methylated according to Hartman and Lago [41] and analysed as described by method Ce 1f-96 from AOCS [42]. Tridecanoic acid was used as an internal standard. An HP 5890 Series II gas chromatograph (Hewlett-Packard, Palo Alto, CA, USA) equipped with a flame ionization detector (FID) and a split injector was used. Separation was done in a capillary fused silica column (100 m × 0.25 mm × 0.2 μm, Agilent J&W GC Columns, Palo Alto, CA, USA) at 130 °C (isothermal). Hydrogen set at a flow rate of 1.5 mL/min was the carrier gas. The injection temperature was 270 °C, and the detector temperature was 280 °C.

#### 3.2.5. Tocopherols

Tocopherol quantification was carried out as described by Ce 8-89 official method from AOCS [43]. A normal phase high performance liquid chromatography (HPLC) was utilized. The HPLC system consisted of the LC-6AD, equipped with a fluorescence detector RF-10A_XL_, automatic interjector SIL-10AF (Shimadzu Scientific Instruments Inc., Columbia, MD, USA), Shimadzu software CLASS-VP and column (Lichrospher Si60, 5 μm, 25 cm × 4 mm i.d., E. Merck, Darmstadt, Germany) connected to a LiChroCART guard column (LiChrospher Si 60, 5 μm, 4 cm × 4 mm i.d., E. Merck, Darmstadt, Germany). The excitation and emission wavelengths for the fluorescent determination of tocopherol isomers were 290 and 330 nm, respectively. Standards of α, γ and δ-tocopherol (Sigma-Aldrich, St Louis, MO, USA) and rac-β-tocopherol (Supelco, Bellefonte, PA, USA) were utilized for the calibration curve. The standards were prepared right before the analysis and corrected as recommended by the official method using a UV mini 1240 Shimadzu spectrophotometer (Shimadzu, Tokyo, Japan). Ten microliters of sample extract or of tocopherol standard solution were injected per run. An isocratic mobile phase comprising 99:1 (v/v) isopropanol in hexanes at a flow rate of 1.0 mL/min was utilized.

#### 3.2.6. Oil Stability Index

The induction period was determined by official method Cd12b-92 from AOCS [44]. A sample of 5.00 g of oil was heated at 110 °C under a dry airflow of 9 L/h in a tube inserted into a 743 Rancimat equipment (Metrohm Corporation, Herisau, Switzerland). The induction period was expressed as hour (h).

#### 3.2.7. UV Absorption

This analysis determines the presence of primary and secondary oxidation products, such as conjugated dienes and trienes, aldehydes and ketones. The specific absorbances at 232 and 270 nm were determined using a UV mini 1240 Shimadzu spectrophotometer (Shimadzu, Tokyo, Japan) following the method Ch 5-91 from AOCS [45].

#### 3.2.8. Statistical Analysis

A completely randomized design with three replicates per treatment was used. Analysis of variance and the Tukey test (*p* < 0.05) were performed with SAS software and correlation analysis were carried out with ASSISTAT 7.6 program.

## 4. Conclusions

As expected a negative correlation was found between irradiation and tocopherol contents as well as irradiation and induction period. Positive correlation was found between gamma radiation and oxidation products as well as the induction period and tocopherol contents. Just moderated decrease in tocopherols was found with storage time. Furthermore, α-tocopherol was the most gamma radiation sensitive. In relation to tocopherol contents, peeled samples were the least recommended feedstock, while in-shell and blanched samples were the best. Gamma radiation and storage time increased the oxidation products of the peanuts. In-shell samples presented the least oxidative damage. Thus, for gamma irradiated peanuts, in-shell samples are the best feedstock.

## Figures and Tables

**Table 1:** Proximate composition 1 (g/100) of dry sample.

Component	Peeled	In-shell 2	Blanched
Ash	2.11 ± 0.07 a 3	2.12 ± 0.05 a	1.90 ± 0.16 a
Lipid	46.92 ± 1.66 a	46.48 ± 4.44 a	47.33 ± 2.77 a
Protein	22.25 ± 1.58 a	20.34 ± 1.52 a	22.80 ± 0.88 a
Fiber	21.23 ± 2.32 a	25.88 ± 2.20 a	22.11 ± 0.76 a
Carbohydrates	5.19 ± 1.78 a	7.49 ± 2.23 a	5.86 ± 2.51 a

1 Data represent the mean of triplicate analysis for each sample ± standard deviations;

2 In-sheel peanuts were hand peeled by hands before the analysis;

3 Means with the same letters within a row are not statistically different by Tukey’s multiple test (*p* < 0.05).

**Table 2:** Fatty acid compositions of the peanut samples (g/100 g) 1.

Fatty acids	Peeled	In-shell	Blanched
C16:0	13.06 ± 0.53 ab 3	13.33 ± 0.02 a	12.14 ± 0.54 b
C18:0	1.54 ± 0.01 b	1.47 ± 0.03 c	2.08 ± 0.00 a
C18:1	46.68 ± 0.09 a	44.94 ± 0.26 b	44.92 ± 0.23 b
C18:2	35.40 ± 0.09 b	37.10 ± 0.32 a	37.63 ± 0.22 a
C20:0	0.59 ± 0.04 a	0.52 ± 0.03 a	0.61 ± 0.05 a
C22:0	0.88 ± 0.04 a	0.77 ± 0.03 b	0.73 ± 0.05 b
C22:1	1.28 ± 0.18 a	1.45 ± 0.46 a	1.32 ± 0.33 a
C24:0	0.57 ± 0.10 a	0.43 ± 0.11 a	0.56 ± 0.13 a
O/L 2	1.32 ± 0.00 a	1.21 ± 0.00 b	1.19 ± 0.01 b
SFA	16.64 ± 0.36 a	16.52 ± 0.12 a	16.12 ± 0.32 a
PUFA	35.40 ± 0.09 b	37.10 ± 0.32 a	37.63 ± 0.22 a
MUFA	47.96 ± 0.27 a	46.38 ± 0.20 b	46.24 ± 0.10 b

1 Data represent the mean of triplicate analysis for each sample ± standard deviations;

2 O/L = oleic/linoleic ratio, SFA = saturated fatty acids, PUFA = polyunsaturated fatty acids, MUFA = monounsaturated fatty acids;

3 Means with the same letters within a row are not statistically different by Tukey’s multiple test (*p* < 0.05).

**Table 3:** Tocopherol contents of the peanut samples 1 (mg/100 g).

Tocopherols	Peanuts	T 2	0.0 kGy	5.0 kGy	7.5 kGy	10.0 kGy
α-Tocopherol	Peeled	0	19.59 ± 0.33 A 3 a 4	7.61 ± 0.31 A b	6.13 ± 0.15 A c	7.82 ± 0.13 A b
		3	18.23 ± 0.71 B a	5.86 ± 0.60 B b	5.13 ± 0.53 B b	6.39 ± 0.98 AB b
		6	18.14 ± 0.46 B a	6.93 ± 0.53 AB b	6.39 ± 0.11 A b	5.92 ± 0.39 B b

	In-shell	0	19.13 ± 0.64 A a	10.70 ± 0.48 A b	10.50 ± 0.34 A b	10.83 ± 0.10 A b
		3	17.81 ± 0.87 AB a	9.61 ± 0.44 A b	10.34 ± 0.17 A b	10.65 ± 0.46 A b
		6	16.05 ± 1.25 B a	9.15 ± 0.93 A b	9.78 ± 0.44 A b	10.22 ± 0.63 A b

	Blanched	0	11.75 ± 1.46 A a	7.52 ± 0.19 A b	7.43 ± 0.11 A b	7.03 ± 0.44 A b
		3	11.88 ± 0.97 A a	7.18 ± 0.41 A b	6.83 ± 0.14 A b	6.88 ± 0.49 A b
		6	12.02 ± 0.11 A a	6.29 ± 0.76 A b	6.59 ± 0.55 A b	7.77 ± 0.62 A b

β-Tocopherol	Peeled	0	0.55 ± 0.01 A a	0.49 ± 0.01 A b	0.36 ± 0.00 A d	0.39 ± 0.01 A c
		3	0.54 ± 0.03 AB a	0.45 ± 0.01 AB b	0.36 ± 0.03 A c	0.37 ± 0.01 AB c
		6	0.50 ± 0.00 B a	0.44 ± 0.02 B b	0.37 ± 0.01 A c	0.34 ± 0.02 B c

	In-shell	0	0.55 ± 0.03 A a	0.52 ± 0.02 A a	0.44 ± 0.01 A b	0.46 ± 0.01 A b
		3	0.54 ± 0.03 A a	0.47 ± 0.01 B b	0.45 ± 0.03 A b	0.47 ± 0.02 AB b
		6	0.47 ± 0.02 B a	0.47 ± 0.00 B a	0.44 ± 0.00 A a	0.44 ± 0.03 B a

	Blanched	0	0.56 ± 0.01 A a	0.54 ± 0.02 A ab	0.52 ± 0.01 A ab	0.50 ± 0.03 A b
		3	0.57 ± 0.03 A a	0.50 ± 0.00 AB b	0.50 ± 0.02 A b	0.49 ± 0.03 A b
		6	0.57 ± 0.02 A a	0.47 ± 0.03 B b	0.47 ± 0.01 A b	0.51 ± 0.01 A b

γ-Tocopherol	Peeled	0	24.21 ± 0.20 A a	18.51 ± 0.48 A b	13.06 ± 0.33 B d	15.56 ± 0.30 A c
		3	23.44 ± 0.85 AB a	17.93 ± 0.04 AB b	12.13 ± 0.24 C d	14.27 ± 0.17 B c
		6	22.66 ± 0.45 B a	17.47 ± 0.33 B b	14.50 ± 0.25 A c	12.53 ± 0.54 C d

	In-shell	0	24.45 ± 0.78 A a	21.03 ± 1.08 A b	18.63 ± 0.78 A c	19.32 ± 0.09 A bc
		3	24.06 ± 1.19 AB a	18.99 ± 0.22 AB b	18.98 ± 0.78 A b	19.47 ± 0.64 A b
		6	21.44 ± 1.41 B a	17.36 ± 1.03 B b	17.41 ± 0.89 A b	18.78 ± 0.29 A b

	Blanched	0	23.74 ± 1.26 A a	20.92 ± 0.29 A b	19.85 ± 0.42 A b	18.88 ± 1.16 A b
		3	23.06 ± 1.47 A a	19.92 ± 1.02 AB b	19.71 ± 0.51 A b	17.89 ± 0.61 A b
		6	22.85 ± 0.26 A a	18.64 ± 1.05 B b	19.14 ± 1.11 A b	19.47 ± 0.11 A b

δ-Tocopherol	Peeled	0	1.44 ± 0.01 Aa	1.00 ± 0.03 A b	0.86 ± 0.02 A c	0.92 ± 0.04 A bc
		3	0.96 ± 0.03 B a	0.93 ± 0.01 B a	0.71 ± 0.02 B c	0.79 ± 0.04 B b
		6	0.87 ± 0.02 C a	0.83 ± 0.01 C a	0.82 ± 0.02 A a	0.73 ± 0.04 B b

	In-shell	0	1.07 ± 0.03 A a	0.99 ± 0.05 A ab	0.92 ± 0.05 A b	0.93 ± 0.01 A b
		3	0.98 ± 0.05 A a	0.88 ± 0.03 A a	0.94 ± 0.01 A a	0.91 ± 0.04 A a
		6	0.85 ± 0.06 B a	0.84 ± 0.08 A a	0.86 ± 0.08 A a	0.80 ± 0.15 A a

	Blanched	0	1.19 ± 0.04 A a	1.16 ± 0.01 A a	1.15 ± 0.01 A a	1.16 ± 0.02 A a
		3	1.13 ± 0.09 A a	1.13 ± 0.06 A a	1.14 ± 0.04 A a	1.09 ± 0.06 A a
		6	1.14 ± 0.08 A a	1.02 ± 0.10 A a	1.12 ± 0.02 A a	1.13 ± 0.03 A a

Total-tocopherol	Peeled	0	45.80 ± 0.52 A a	27.61 ± 0.78 A b	20.42 ± 0.46 B d	24.69 ± 0.43 A c
		3	43.17 ± 1.61 AB a	25.17 ± 0.62 B b	18.33 ± 0.59 C d	21.83 ± 1.06 B c
		6	42.16 ± 0.93 B a	25.67 ± 0.72 B b	22.08 ± 0.34 A c	19.52 ± 0.95 C d

	In-shell	0	45.19 ± 1.45 A a	33.23 ± 1.59 A b	30.49 ± 1.18 A b	31.53 ± 0.17 A b
		3	43.39 ± 2.13 AB a	29.95 ± 0.54 AB b	30.71 ± 0.69 A b	31.50 ± 1.16 A b
		6	38.80 ± 2.62 B a	27.82 ± 1.77 B b	28.50 ± 1.21 A b	30.23 ± 0.66 A b

	Blanched	0	37.23 ± 2.71 A a	30.14 ± 0.49 A b	28.94 ± 0.54 A b	27.57 ± 1.62 A b
		3	36.65 ± 2.42 A a	28.73 ± 1.49 AB b	28.18 ± 0.71 A b	26.35 ± 1.07 A b
		6	36.58 ± 0.31 A a	26.41 ± 1.76 B b	27.32 ± 1.54 A b	28.87 ± 0.50 A b

1 Data represent the mean of triplicate analysis for each sample ± standard deviations;

2 T = Storage time in months;

3 Means with the same small letter within a row are not statistically different by Tukey’s multiple test (*p* < 0.05);

4 Means with the same capital letters within a column are not statistically different by Tukey’s multiple test (*p* < 0.05).

**Table 4:** Pearson’s correlation between gamma radiation and tocopherol contents.

Tocopherols	Peanuts	Time zero	Third month	Sixth month
α-Tocopherol	Peeled	−0.87 **	−0.85 **	−0.91 **
	In-shell	−0.87 **	−0.81 **	−0.78 **
	Blanched	−0.87 **	−0.88 **	−0.73 **

β-Tocopherol	Peeled	−0.91 **	−0.93 **	−0.97 **
	In-shell	−0.85 **	−0.73 **	−0.62 *
	Blanched	−0.82 **	−0.79 **	−0.62 *

γ-Tocopherol	Peeled	−0.90 **	−0.91 **	−0.99 **
	In-shell	−0.90 **	−0.80 **	−0.80 **
	Blanched	−0.92 **	−0.90 **	−0.73 **

δ-Tocopherol	Peeled	−0.91 **	−0.76 **	−0.86 **
	In-shell	−0.84 **	−0.47 ns	−0.14 ns
	Blanched	−0.53 ns	−0.21 ns	−0.01 ns

Total-Tocopherols	Peeled	−0.90 **	−0.91 **	−0.97 **
	In-shell	−0.90 **	−0.81 **	−0.73 **
	Blanched	−0.91 **	−0.91 **	−0.73 **

** significant (*p* < 0.01);

* significant (*p* < 0.05);

ns non significant.

**Table 5:** Induction period (h) of the crude peanut oils 1.

Peanuts	Dose (kGy)	Induction period 1 (h)		

		Time zero	Third month	Sixth month
Peeled	0.0	10.29 ± 0.06 A 2 a 3	9.66 ± 0.11 B a	9.80 ± 0.11 B a
	5.0	8.59 ±0.25 AB b	8.46 ± 0.04 B b	8.80 ± 0.04 A b
	7.5	8.26 ± 0.02 A c	7.89 ± 0.07 B c	8.24 ± 0.09 A c
	10.0	8.25 ± 0.03 A c	8.30 ± 0.04 A b	7.47 ± 0.04 B d

In-shell	0.0	10.25 ± 0.02 A a	9.12 ± 0.05 B a	10.37 ± 0.04 A a
	5.0	9.14 ± 0.03 B b	8.46 ± 0.11 C c	9.82 ± 0.05 A b
	7.5	9.11 ± 0.08 B b	8.91 ± 0.11 B ab	9.49 ± 0.01 A c
	10.0	8.68 ± 0.08 B c	8.74 ± 0.05 AB bc	8.96 ± 0.07 A d

Blanched	0.0	10.74 ± 0.06 B a	10.92 ± 0.13 AB a	11.12 ± 0.02 A a
	5.0	9.92 ± 0.03 B b	10.14 ± 0.02 A b	10.20 ± 0.03 A b
	7.5	9.58 ± 0.10 B c	9.63 ± 0.11 B c	9.90 ± 0.03 A c
	10.0	9.46 ± 0.12 B c	9.74 ± 0.01 A c	9.62 ± 0.03 AB d

1 Data represent the mean of triplicate analysis for each sample ± standard deviations;

2 Means with the same capital letters within a row are not statistically different by Tukey’s multiple test (*p* < 0.05);

3 Means with the same small letter within a column are not statistically different by Tukey’s multiple test (*p* < 0.05).

**Table 6:** Pearson’s correlation between gamma radiation and induction period and between total tocopherol and induction period.

Related variables	Peanut	Time zero	Third month	Sixth month
Irradiation × Induction period	Peeled	−0.93 **	−0.87 **	−0.99 **
	In-shell	−0.95 **	−0.43 ns	−0.98 **
	Blanched	−0.97 **	−0.93 **	−0.99 **

Total tocopherol × Induction period	Peeled	0.97 **	0.98 **	0.93 **
	In-shell	0.93 **	0.72 **	0.64 *
	Blanched	0.92 **	0.86 **	0.81 **

** significant (*p* < 0.01);

* significant (*p* < 0.05);

ns non significant.

**Table 7:** Primary oxidation products (E_232nm_) 1.

Peanut	Dose (kGy)	E_232nm_		

		Time zero	Third month	Sixth month
Peeled	0.0	1.016 ± 0.028 B 2 d 3	1.443 ± 0.053 A c	1.062 ± 0.010 B d
	5.0	1.594 ± 0.092 A c	1.701 ± 0.072 A b	1.191 ± 0.012 B c
	7.5	1.955 ± 0.079 A b	1.880 ± 0.078 A b	1.349 ± 0.019 B b
	10.0	2.287 ± 0.027 B a	2.581 ± 0.157 A a	1.392 ± 0.010 C a

In-shell	0.0	1.502 ± 0.004 A d	1.430 ± 0.002 B bc	1.185 ± 0.027 C d
	5.0	1.650 ± 0.067 A c	1.321 ± 0.020 B b	1.289 ± 0.004 B c
	7.5	1.872 ± 0.043 A b	1.461 ± 0.033 B b	1.398 ± 0.011 B b
	10.0	2.173 ± 0.019 A a	1.610 ± 1.610 B a	1.443 ± 0.008 C a

Blanched	0.0	3.622 ± 0.001 A c	3.643 ± 0.080 A b	3.032 ± 0.102 B a
	5.0	3.508 ± 0.004 B c	3.815 ± 0.053 A b	2.758 ± 0.005 C bc
	7.5	3.946 ± 0.126 B b	4.403 ± 0.033 A a	2.716 ± 0.001 C c
	10.0	4.513 ± 0.008 A a	4.408 ± 0.178 A a	2.852 ± 0.003 B b

1 Data represent the mean of triplicate analysis for each sample ± standard deviations;

2 Means with the same capital letters within a row are not statistically different by Tukey’s multiple test (*p* < 0.05);

3 Means with the same small letter within a column are not statistically different by Tukey’s multiple test (*p* < 0.05).

**Table 8:** Secondary oxidation products (E_270nm_) 1.

Peanut	Dose (kGy)	E_270nm_		

		Time zero	Third month	Sixth month
Peeled	0.0	0.024 ± 0.001 C 2 c 3	0.448 ± 0.025 A b	0.095 ± 0.040 B b
	5.0	0.102 ± 0.003 C b	0.561 ± 0.014 A b	0.168 ± 0.030 B ab
	7.5	0.186 ± 0.001 B a	0.573 ± 0.044 A b	0.231 ± 0.009 A a
	10.0	0.196 ± 0.007 B a	0.884 ± 0.085 A a	0.235 ± 0.029 B a

In-shell	0.0	0.048 ± 0.001 B d	0.149 ± 0.018 A b	0.055 ± 0.018 B c
	5.0	0.087 ± 0.007 B c	0.140 ± 0.008 A b	0.142 ± 0.016 A b
	7.5	0.105 ± 0.005 C b	0.224 ± 0.017 A a	0.194 ± 0.007 B a
	10.0	0.119 ± 0.006 C a	0.252 ± 0.012 A a	0.174 ± 0.015 B ab

Blanched	0.0	0.130 ± 0.001 B d	0.536 ± 0.021 A a	0.184 ± 0.038 B c
	5.0	0.182 ± 0.003 C c	0.591 ± 0.047 A a	0.291 ± 0.011 B b
	7.5	0.208 ± 0.006 C b	0.631 ± 0.034 A a	0.335 ± 0.021 B ab
	10.0	0.421 ± 0.008 B a	0.650 ± 0.078 A a	0.364 ± 0.025 B a

1 Data represent the mean of triplicate analysis for each sample ± standard deviations;

2 Means with the same capital letters within a row are not statically different by Tukey’s multiple test (*p* < 0.05);

3 Means with the same small letter within a column are not statistically different by Tukey’s multiple test (*p* < 0.05).

**Table 9:** Pearson’s correlation between gamma radiation and oxidation products.

Related variables	Peanut	Time zero	Third month	Sixth month
Irradiation × Primary oxidation products	Peeled	0.99 **	0.89 **	0.98 **
	In-shell	0.94 **	0.57 ns	0.98 **
	Blanched	0.79 **	0.89 **	0.63 *
Irradiation × Secondary oxidation products	Peeled	0.98 **	0.84 **	0.90 **
	In-shell	0.99 **	0.82 **	0.90 **
	Blanched	0.84 **	0.73 **	0.95 **

** significant (*p* < 0.01);

* significant (*p* < 0.05);

ns non significant.
